# Treatise on the Diseases of Old Age

**Published:** 1849-09

**Authors:** 


ART. IV.—*A Practical Trealies on the Domestic Management and most Important Diseases of Advanced Life*—With an Appendix, containing a series of cases illustrative of a new and successful mode of treating Lumbago, and after forms of Chronic Rheumatism, Sciatica, and other Neuralgic Affections, and certain forms of Paralysis.—By GEORGE E. DAY, M. D., Fellow of the Royal College of Physicians, and Physician to the Western General Dispensary, Philadelphia: pp 226: 8 vo: Lea & Blanchard. (From the Publishers. For sale by S. C. Griggs & Co.

It is the object of the Profession of Medicine, not only to cure the diseases and relieve the sufferings of youth and Middle age; but, when our art cannot avert the destruction which at last overtakes the human machine, to smooth the descending path, and render less terrible the sufferings attendant on the slowly approaching but certain dissolution. For this purpose Dr. Day has written this excellent treatise, for which are due the thanks of the Profession and all Octogenarians: The work contains much curious learning and research to entertain and interest the prófessional scholar, as well as a large amount of valuable information to attract the attention of the practical physician. We heartily recommend it to our readers, and regret our inability to furnish them an analysis of its contents. M.

